# 1149. Resistance Analyses of Patient Viral Samples from the Remdesivir Phase 3 Adaptive COVID-19 Treatment Trial-1 (ACTT-1)

**DOI:** 10.1093/ofid/ofac492.987

**Published:** 2022-12-15

**Authors:** Charlotte Hedskog, Lauren Rodriguez, John H Beigel, Walla Dempsey, Alexander L Greninger, Pavitra Roychoudhury, Meei-Li Huang, Keith R Jerome, Linhui Hao, Renee Ireton, Michael Gale, Jiani Li, Jason Perry, Dong Han, Gregory Camus, Danielle P Porter

**Affiliations:** Gilead Sciences Inc, Foster City, California; Gilead Sciences Inc, Foster City, California; The National Institute of Allergy and Infectious Diseases, National Institutes of Health, Rockville, Maryland; The National Institute of Allergy and Infectious Diseases, National Institutes of Health, Rockville, Maryland; University of Washington, Seattle, Washington; University of Washington, Seattle, Washington; University of Washington, Seattle, Washington; University of Washington, Seattle, Washington; University of Washington, Seattle, Washington; University of Washington, Seattle, Washington; University of Washington, Seattle, Washington; Gilead Sciences Inc, Foster City, California; Gilead Sciences Inc, Foster City, California; Gilead Sciences Inc, Foster City, California; Gilead Sciences Inc, Foster City, California; Gilead Sciences Inc, Foster City, California

## Abstract

**Background:**

Remdesivir (RDV) is a broad-spectrum nucleotide analog prodrug approved for the treatment of COVID-19 in non-hospitalized and hospitalized adult as well as pediatric patients with clinical benefit demonstrated in multiple Phase 3 trials. Here we present SARS-CoV-2 resistance analyses from the Phase 3 ACTT-1 placebo-controlled clinical trial in hospitalized adults.

**Methods:**

Oro- or nasopharyngeal swab samples in ACTT-1 study were collected on Day 1, 3, 5, 8, 11, 15, and 29. All participants with >80^th^ and 50% of participants with < 20^th^ percentile of cumulative viral shedding underwent resistance analysis in both the RDV and placebo arm. The SARS-CoV-2 genome was sequenced using next generation sequencing. Phenotyping was conducted using virus isolation from clinical samples or generation of select site-directed mutants (SDMs) in a SARS-CoV-2 replicon system.

**Results:**

The majority of the sequencing data were obtained from participants with 80^th^ percentile of cumulative viral shedding from the RDV and placebo arms as shown in Table 1. Among participants with both baseline and postbaseline sequencing data, emergent substitutions in nsp12 were observed in 12 of 31 participants (38.7%) treated with RDV and 12 of 30 participants (40.0%) in the placebo arm. The nsp12 substitutions that emerged in the RDV arm were only observed in one participant each, and the majority were present as mixtures with wildtype sequence. The following nsp12 mutations emerged in the RDV treatment group and were successfully phenotyped as clinical isolates or SDMs with low to no fold change in RDV susceptibility: A16V (0.8-fold), P323L+V792I (2.2-fold), C799F (2.5-fold), K59N (1.0-fold), and K59N+V792I (3.4-fold). V792I and C799F were identified previously *in vitro* in resistance selection experiments (Stevens Sci Transl Med 2022). In addition, for D684N and V764L identified in the RDV arm, the recovery of neither clinical isolates nor SDMs for phenotypic analysis were successful.

**Conclusion:**

The similar rate of emerging nsp12 substitutions in participants treated with RDV compared to placebo and the minimal to no change in RDV susceptibility among the treatment-emergent nsp12 substitutions support a high barrier to RDV resistance development in COVID-19 patients.

**Figure:**
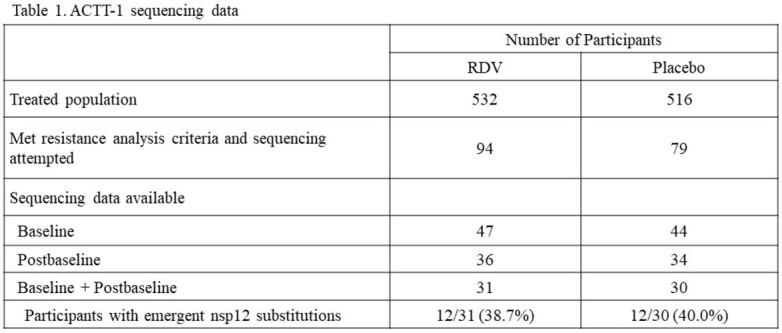

**Disclosures:**

**Charlotte Hedskog, PhD**, Gilead Sciences: Stocks/Bonds **Lauren Rodriguez, PhD**, Gilead: Stocks/Bonds **Alexander L. Greninger, MD, PhD**, Abbott: Contract Testing|Cepheid: Contract Testing|Gilead: Grant/Research Support|Gilead: Contract Testing|Hologic: Contract Testing|Merck: Grant/Research Support|Novavax: Contract Testing|Pfizer: Contract Testing **Jiani Li, PhD**, Gilead Sciences: Stocks/Bonds **Jason Perry, PhD**, Gilead Sciences: Employee|Gilead Sciences: Stocks/Bonds **Dong Han, MS**, Gilead Sciences: Stocks/Bonds **Gregory Camus, PhD**, Gilead Sciences: Employee and shareholder|Gilead Sciences: Stocks/Bonds **Danielle P. Porter, PhD**, Gilead Sciences: Employee|Gilead Sciences: Stocks/Bonds|Gilead Sciences: Stocks/Bonds.

